# Nest Architecture and Colony Growth of *Atta bisphaerica* Grass-Cutting Ants

**DOI:** 10.3390/insects11110741

**Published:** 2020-10-29

**Authors:** Adriano Pimentel Farias, Roberto da Silva Camargo, Kátia Kaelly Andrade Sousa, Nadia Caldato, Luiz Carlos Forti

**Affiliations:** Department of Vegetal Protection, School of Agronomical Sciences, São Paulo State University, Botucatu, SP CEP 18603-970, Brazil; camargobotucatu@yahoo.com.br (R.d.S.C.); katiakaelly@gmail.com (K.K.A.S.); nacbiol@gmail.com (N.C.); luiz.forti@unesp.br (L.C.F.)

**Keywords:** worker number, fungus garden growth, nest development, leaf-cutting ants

## Abstract

**Simple Summary:**

*Atta bisphaerica* grass-cutting ants build giant nests with hundreds or thousands of large chambers. Here, we assessed whether the total volume of fungus chambers and other nest parameters grow at close or similar proportions to worker numbers in the *A. bisphaerica* colony. The fungus garden biomass, external area, and total fungus chamber volume increased at rates similar to that of worker numbers. The total volume of chambers, and external area increased at a similar proportion to that of the number of workers, probably due to the fungus garden allocation needs of the colony. On the other hand, the number of fungus chambers, number of entrance holes, and nest depth increased more slowly than the number of workers. Thus, this study demonstrates that *A. bisphaerica* nest development regarding the total volume of chambers is similar to the increase in the number of colony workers.

**Abstract:**

*Atta* grass-cutting ants (Formicidae: Myrmicinae: Attini) are found in the Cerrado biome and build giant nests with hundreds or thousands of large chambers. We assessed for *Atta bisphaerica* grass-cutting ants whether the total volume of fungus chambers and other nest parameters grow at close or similar proportions to worker numbers in the colony. Data on fungus garden biomass, population, external area, number of entrance holes, number of fungus chambers, total fungus chambers volume, and nest depth were obtained during colony growth/nest development. Our results reveal that the fungus garden biomass, external area, and total fungus chamber volume increased at rates similar to the increase in the number of workers. The total volume of chambers, and external area increased at a similar proportion to the increase in number of workers, probably due to the fungus garden allocation needs of the colony. The number of fungus chambers, number of entrance holes, and nest depth increased less than 4-fold for every 10-fold increase in the worker number. In addition, the height of the fungus chambers increased as the width increased, a pattern not observed for tunnel height and length, and the chamber volume increased according to worker number. Thus, this study demonstrates for *A. bisphaerica* that nest development in terms of chamber volume is similar to the increase in number of colony workers, and this contributes to a better understanding of *Atta* nest architecture.

## 1. Introduction

Nests, burrows, traps, galls, and several structures that animals build are essential for the animal’s survival and are products of natural selection [1,2]. Some species depend on the availability of building materials (e.g., fishes of the family Gasterosteidae that use fibrous threads embedded in nests, and insects of the order Trichoptera that need sediment to form their burrows) [3,4]. Spiders and antlions build traps based on self-secretions from the body (like silk) or on the arrangement of sand particles, essential for prey capture [5,6]. Ant species of the genera *Pseudomyrmex* and *Camponotus* build nests on trees from preformed cavities and organic materials [7,8]. In the soil, species of *Pheidole*, *Odontomachus*, and *Pogonomyrmex* dig less than a hundred small chambers [9,10,11]. However, leaf-cutting ants, especially of the *Atta* genus, dig complex subterranean nests with hundreds/thousands of large chambers [12,13,14].

For leaf-cutting ants (*Atta*), it was proposed that the high mortality of semi-claustral queens was sufficient to support the natural selection of fully claustral queens [15]. Claustral queens are larger in size and rich in the lipids needed for reproduction and nest foundation, at the expense of their energy reserves [15,16,17]. However, these queens are energetically expensive for the mother colony, which needs to store large amounts of food resources [15]. Thus, the colony must continuously adjust the nest to accommodate food resources (fungus garden) with the aim to produce thousands of alates [18].

The availability of food resources could influence an increase in worker numbers and fungus gardens for colony growth [19,20]. Food resources seem to be abundant when grass-cutting ants such as *Atta bisphaerica* Forel 1908 inhabit pasture environments; however, few grass species are selected for foraging [21]. Thus, the nests built by *A. bisphaerica* can be considered functionally versatile extensions of the colony and are a direct way of studying how these structures are adjusted by ants (workers) [22]. Nests of *A. bisphaerica* are characterized by a lower number of fungus chambers when compared to species that cut dicotyledons, such as *A. sexdens* [23]. In addition, they make nests that have a large number of entrance holes for workers to explore the environment [21]. On the other hand, *A. bisphaerica* do not make specialized chambers for waste depositing (waste chambers), as is the case for the grass-cutting ant *A. capiguara* [14].

Previous studies showed the external area pattern and the number of chambers and tunnels in mature *A. bisphaerica* nests; they also showed the number of entrance holes and tunnels that were influenced by the availability of food resources [12,21]. Furthermore, the nest development pattern was obtained from the nests of different ages [23]. Although these studies mention the nest architecture of grass-cutting ants, they are based only on molded nests and draw no relation to colony parameters (number of workers and fungus garden biomass). In *Atta*, the mature colonies are large and contain from several thousand to over a million workers [24].

In ants in general, the nest size is related to the number of workers, given that they build their nest in some proportion to their needs [24,25]. However, studies showed that the nest parameters of *Pogonomyrmex* and *Aphaenogaster* ants (e.g., total area of chambers) increased more slowly than the worker population [25,26]. In this context, we hypothesized for grass-cutting ants that the total volume of fungus chambers, and even other nest parameters, grows at close or similar proportions to that of worker number growth in the colony (because of the nest space that the fungus garden occupies). Thus, the present study provides a description of the nest architecture development for the grass-cutting ant *A. bisphaerica* according to the number of workers, and it contributes to a better understanding of *Atta* nest architecture.

## 2. Materials and Methods

### 2.1. Experimental Area

This study was conducted at Lageado Experimental Farm (22°50′47′′ S; 48°26′01′′ W) and Santana Farm (22°50′44′′ S; 48°26′12′′ W), Botucatu, São Paulo, Brazil. On the farms, the pasture was formed using *Brachiaria* spp., with patches of *Paspalum* spp., the preferred nesting places of grass-cutting ants.

### 2.2. Study Colonies and Nests

*A. bisphaerica* nests were mapped since their nuptial flight in October 2014, according to Forti et al. [23]. After 2, 8, 20, 32, 44, and 56 months of nest foundation, 3, 4, 4, 3, 1, and 1 colonies of each age, respectively, were studied. Before the excavation, the external area of the nest was measured by the width and length of the area formed by all the mounds of loose soil [27]. In addition, the entrance holes in each nest were counted and marked, and talc was sprinkled in each hole as a guide for locating the chambers and deeper tunnels. The nests were manually excavated by opening trenches at intervals of 1 m deep to collect the biomass of each fungus chamber (fungus garden and population), as well as to measure the dimensions of the chambers and tunnels [12,23].

### 2.3. Population and Symbiont Fungus Garden Biomass

The fungus garden and population were collected in plastic pots ranging from 0.5 to 15 L, after opening each chamber. The fungus garden was immediately taken to the laboratory to obtain a fresh weight and weighing 20% to estimate the dry weight. Drying was carried out in an oven with air circulation at 50 °C for 24 h, and the total fungus garden dry biomass of each nest was obtained by extrapolating dry mass values to 100%. The population of each nest and chamber was conserved in 70% alcohol, and 10% of the collected volume was used to count the number of workers. Workers were separated and counted, and the total population of each nest was obtained by extrapolating the number to 100% volume.

### 2.4. Architecture

Fungus chambers found according to excavation were counted and their width, height, and distance to the surface were measured. The foraging tunnels and tunnels that connect the chambers were measured in width, height, length, and depth. The tunnels that connect chambers were counted and measured in nests up to 32 months, due to a large number of these structures in older nests. In the nests aged 44 and 56 months, the dimensions of the foraging tunnels were obtained from three tunnels in each quadrant of the nest.

The volume of each fungus chamber was estimated based on the cylinder volume. However, as the chambers are rounded, a correction factor was used for calculating the volume, V = πr^2^ (ch + r0.67), in which ‘r’ is chamber base radius and ‘ch’ is cylinder height, measured by subtracting the maximum height of the chamber from its radius, ch = h − r [23]. The total volume of the fungus chambers at each age was obtained by the sum of each chamber volume.

### 2.5. Statistical Analyses

Linear regression analyses of log-transformed variables were used to determine the fungus garden biomass growth and nest architecture development in relation to the increase in number of colony workers. The nest architecture variables evaluated were external area, number of entrance holes, number of fungus chambers, total fungus chambers volume, and nest depth. Bivariate scaling analyses (log–log) of worker number with (a) fungus garden biomass, (b) number of fungus chambers, (c) number of entrance holes, (d) external area, (e) total fungus chambers volume, and (f) depth of nests were performed to test allometric relationships. The growth in fold number in workers was also used to determine the proportion of growth for every variable. In addition, the dimensions of chambers and tunnels were subjected to regression analysis, and data on architecture parameters, number of workers, and fungus garden biomass according to colony age (2, 8, 20, 32, 44, and 56 months) were subjected to descriptive statistics to obtain the mean, standard deviation, and maximum and minimum values. All analyses were performed with Statistica software version 7.0 (StatSoft, Tulsa, USA) [28].

## 3. Results

The *A. bisphaerica* nests aged 2 months had only one entrance hole, external area around 200 cm^2^, fungus chamber with 160 mL volume, and depth of 18 cm. At 8 months of age, the nests increased to two fungus chambers (total chamber volume around 700 mL), external area to 360 cm^2^, and reached a depth of 0.9 m (Table 1). From 20 to 56 months of age, the number of entrance holes increased from 2 to 132, the number of chambers increased from 2 to 104 (total chamber volume from 6 to 290 L), external area from 0.2 to 42 m^2^, and depth from 1.6 to 3.4 m (Table 1). The colony aged 2 months had around 121 workers and 5 g of fungus garden dry biomass. At 8 months of age, the number of workers increased to around 700 and fungus garden to 21 g. From 20 to 56 months of age, the number of workers increased from around 3000 to 450,000, while fungus garden dry biomass increased from 100 g to 12 kg (Table 1).

**Figure 1:**
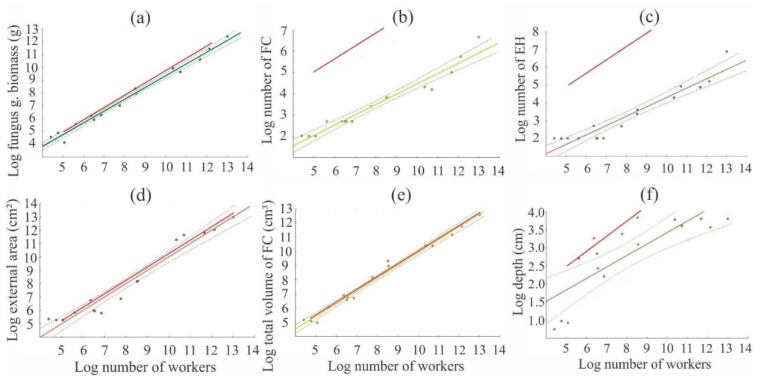
Relationship of *A. bisphaerica* worker number with (**a**) fungus garden biomass, (**b**) number of fungus chambers (FC), (**c**) number of entrance holes (EH), (**d**) external area, (**e**) total fungus chambers volume (FCV), and (**f**) depth of nests. The red line represents the expected slope of 1 (**a**–**e**) or 0.5 (**f**). Values and their 95% confidence intervals are shown (n = 16 colonies/nests).

The fungus garden biomass, external area, and total fungus chamber volume increased at similar rates to the increase in the number of workers (Figure 1; Table 2). Among all variables, the number of fungus chambers, entrance holes, and nest depth had greater variations in the increase rate (Figure 1; Table 2). However, the proportion found between how much the fungus garden and nest factors increased according to the worker number ranged from 0.25:1 (2.5:10) to 1:1 (10:10). The total fungus chamber volume and external area increased 8- and 10-fold, respectively, for every 10-fold increase in the number of workers (Figure 1c,d). The fungus garden biomass increased around 8.3-fold for every 10-fold increase in the number of workers (Figure 1a). In addition, the number of fungus chambers, number of entrance holes, and nest depth increased 3.1-, 3.4-, and 2.5-fold, respectively, for every 10-fold increase in number of workers (Figure 1b,c,f).

**Figure 2:**
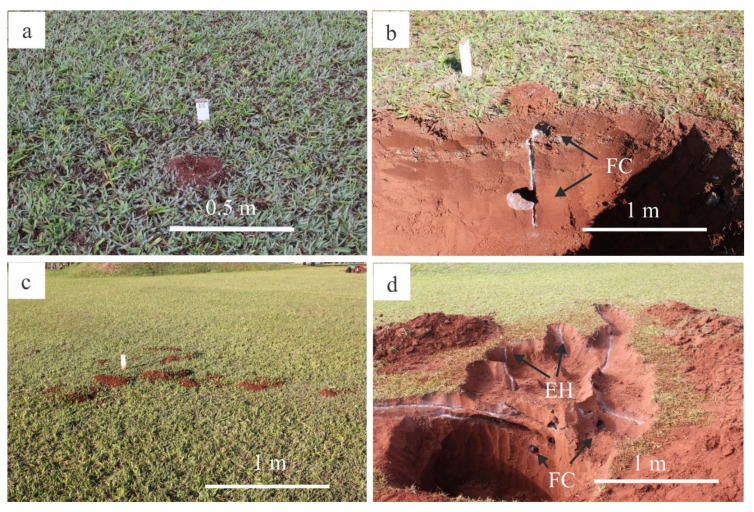
External area and internal architecture of the nests aged 8 months (**a**), (**b**) and 32 months (**c**), (**d**). FC—fungus chambers, EH—entrance holes.

**Figure 3:**
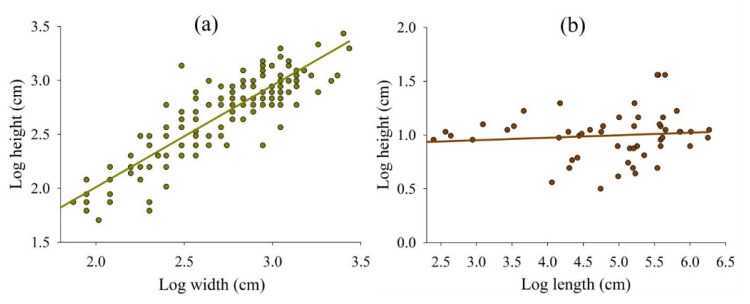
Height of fungus chambers (FC) increased with increase in width (**a**), and height increased with increase in length in foraging tunnels (FT) (**b**). FC: log y = 0.13 + 0.94log x, *R*^2^ = 0.71, *p* <0.001; FT: log y = 0.88 + 0.02log x, *R*^2^ = 0.01, *p* = 0.48.

In addition to vertical growth, the nests grew laterally, and fungus chambers were located in loose soil projections (external area) (Figure 2). Fungus chambers and tunnels, in general, varied in size during nest development. The height of fungus chambers increased as the width increased; however, the chambers were limited in size (Figure 3a). Foraging tunnels were found in nests aged 20 months and over; however, tunnel height did not increase as the length increased (Figure 3b).

**Figure 4:**
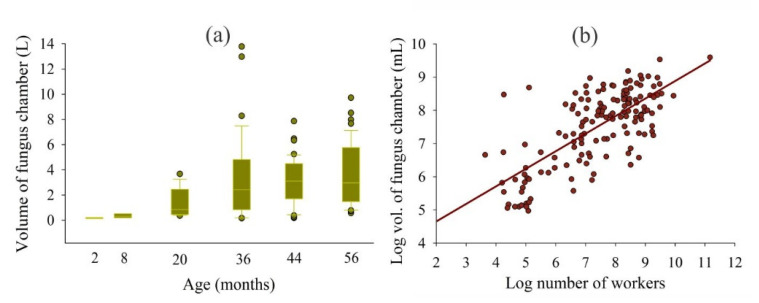
Volume of each fungus chamber by nest age (**a**), and increase in fungus chamber volume with increase in worker number (**b**). Boxes represent between 25 and 75% of the fungus chamber volume, while bars represent between 5 and 95%. Log y = 3.59 + 0.52log x, *R*^2^ = 0.52, *p* <0.001.

The *A. bisphaerica* nests had chambers with a volume ranging from around 200 mL to 14 L (Figure 4a). In young nests (2–8 months old), we found only chambers with a volume less than 1 L. For nests older than 20 months, we found chambers with a volume from less than 1 L to more than 10 L (Figure 4a). In addition, the volume of each chamber increased as the worker numbers increased (Figure 4b).

## 4. Discussion

After the nest is founded by an *Atta* queen, every effort is directed to producing a large number of workers who will take over the activities of nest building, resource collection, and fungus garden growing [24]. Thus, our results show that the development of *A. bisphaerica* fungus garden biomass, total fungus chambers volume, and external area of nests were isometric according to the number of workers (Figure 1; Table 2).

The proportional increase in total fungus chamber volume and external area with the worker number increase is attributed to the colony’s need for space to mainly allocate the fungus garden. Several studies support the hypothesis that the fungus garden and presence of ants in preimaginal stages within the colony are incentives for the workers to expand the space and build new chambers [29,30,31]. An *Atta* chamber is much larger than the documented chambers of other ant genera, and consequently this contributes to a greater total volume of the nest [13,14]. In this study on *A. bisphaerica,* we found chambers up to 14 L volume, although studies with *A. capiguara* revealed chambers with 22 L volume [14]. For the ant *Solenopsis invicta* (which does not grow fungus but can reach thousands of workers similar to *A. bisphaerica*), it was speculated that the population divided into groups and small chambers (e.g., floor area of 5 cm^2^) benefits the colony in communication [32].

The external area (loose soil) of *A. bisphaerica* nests is the result of chambers and tunnels dug to grant access to the environment (Figure 2a,c). For *A. bisphaerica*, soil pellets are deposited in a defined location; thus, loose soil is well spread around the nest [33]. On the other hand, *A. sexdens* and *A. capiguara* do not deposit soil pellets in a defined place, and as they roll, loops of loose soil with greater height are formed close to the nest holes [33]. In *A. capiguara*, the external area is also a result of the digging of waste chambers [14]. As a behavioral adaptation when inhabiting moist and clayey soils, *A. vollenweideri* use soil pellets to form external structures (i.e., ‘turrets’) that assist in nest ventilation [34].

The increase in fungus garden biomass proportional to the worker number increase is possibly an energy strategy for the colony [35,36]. A mature *Atta* colony needs a large fungus biomass for alates production (e.g., an *A. bisphaerica* colony releases around 1690 females and 5360 males annually) [18]. In their nuptial flight, *Atta* queens have maximum internal reserves, with a mass ranging from 400 to 800 mg [37]. Nuptial flight also depends on suitable conditions, and the colony needs to feed the alates for a certain period [18,38]. In addition, the allometry of ants trends toward larger workers as colonies grow, and larger workers have lower metabolisms than smaller workers [24,39].

The increase in the number of chambers and entrance holes at a proportion of less than 4-fold for each 10-fold increase in worker number is attributed to the large chamber size and allocation of thousands of workers and large fungus garden biomass. Our results show that fungus chambers of *A. bisphaerica* grow in dimensions (height as a function of width) and volume increases of around 3-fold for each 10-fold increase in worker number; however, their size is limited and does not exceed 14 L volume (Figure 3 and Figure 4). This occurs because workers adjust expansion up to a certain space, and then they add a new chamber [29,30]. Thus, nest development is based mainly on the enlargement and addition of chambers (increase of 1 to 104 in a period of 56 months). The tunnel dimensions also vary, probably due to the increased number and flow of workers (Figure 3) [23]. However, some tunnels are higher and shorter in length, while others are lower in height and longer (Figure 3).

The 2.5-fold increase in nest depth with each 10-fold increase in worker number certainly occurs because exposed nests in pastures have deep fungus chambers (up to 3.41 m), given that soil temperature is negatively correlated with soil depth [40]. The symbiont fungus and young ant phases need suitable temperature conditions around 25 °C for growth [31]. This arrangement of chambers in the soil certainly interferes with nesting depth, although most chambers are located between 1 and 2 m deep.

## 5. Conclusions

This study demonstrated that the development of *A. bisphaerica* nest structure is isometric according to worker number, with an increase in total volume of chambers at a similar rate to the increase in the number of workers. These findings indicate that larger dimensions of *Atta* nests are not only the result of a large population, as previously thought, but the fungus garden is also a relevant factor. Future studies should be conducted with *Atta* species that nest in forests (closed environments), and potential changes in the communication of these ants that may have arisen from the larger nests should also be investigated.

## Figures and Tables

**Table 1:** External area (EA), number of entrance holes (EH), number of fungus chambers (CF), total fungus chamber volume (FCV), nest depth (Dp), fungus garden biomass (Kg), and worker population (W) of *A. bisphaerica* along with the colony growth/nest development, Botucatu, SP, 2019.

Age (months)		EA (m^2^)	EH	FC	FCV (L)	Dp (m)	FGB (Kg)	W (×1000)
2	Mean	0.020	1.00	1.00	0.161	0.18	0.005	0.121
	SD *	0.0008	0.00	0.00	0.015	0.02	0.002	0.039
	Max.	0.040	1.00	1.00	0.175	0.19	0.006	0.161
	Min.	0.032	1.00	1.00	0.144	0.15	0.003	0.083
8	Mean	0.036	1.00	2.00	0.705	0.96	0.021	0.662
	SD *	0.003	0.00	0.00	0.190	0.26	0.006	0.285
	Max.	0.021	1.00	2.00	0.859	1.25	0.026	0.970
	Min.	0.019	1.00	2.00	0.434	0.67	0.013	0.280
20	Mean	0.220	3.25	4.50	5.822	2.27	0.107	3.315
	SD *	0.150	1.50	1.91	4.465	0.79	0.083	2.242
	Max.	0.352	5.00	6.00	11.050	3.41	0.207	5.203
	Min.	0.086	2.00	2.00	0.980	1.61	0.024	0.587
32	Mean	10.657	15.67	13.00	45.729	3.07	1.267	65.144
	SD *	2.598	4.93	6.08	22.089	0.02	0.683	46.079
	Max.	13.011	19.00	20.00	71.232	3.30	2.042	117.772
	Min.	7.869	10.00	9.00	32.596	2.70	0.752	32.047
44	n = 1	16.342	25.00	42.00	130.988	2.60	4.610	192.611
56	n = 1	42.125	132.00	104.00	291.537	3.30	12.359	444.224

* SD: standard deviation.

**Table 2:** Summary of relationships between nest development parameters and worker number growth in *A. bisphaerica* nests/colonies. F-test; *R*^2^: determination coefficient.

Relationships	Models	F _(1,14)_	*p*-Value	*R* ^2^
Workers (W) vs. Fungus biomass	log y = −2.87 + 0.92log x	918.63	<0.001	0.98
W vs. Fungus chambers (FC)	log y = −2.39 + 0.48log x	262.81	<0.001	0.94
W vs. Entrance holes	log y = −2.98 + 0.53log x	144.33	<0.001	0.90
W vs. External area	log y = −0.02 + 1.00log x	268.14	<0.001	0.95
W vs. Total volume of FC	log y = −0.91 + 0.90log x	907.24	<0.001	0.98
W vs. Depth	log y = 2.25 + 0.31log x	26.84	<0.001	0.63

n = 16 nests/colonies.
